# Association between changes in thyroid hormones and incident type 2 diabetes using joint models of longitudinal and time-to-event data: more than a decade follow up in the Tehran thyroid study

**DOI:** 10.3389/fendo.2024.1475286

**Published:** 2024-12-13

**Authors:** Alireza Amirabadizadeh, Ladan Mehran, Atieh Amouzegar, Samaneh Asgari, Davood Khalili, Fereidoun Azizi

**Affiliations:** ^1^ Endocrine Research Center, Research Institute for Endocrine Sciences, Shahid Beheshti University of Medical Sciences, Tehran, Iran; ^2^ Prevention of Metabolic Disorders Research Center, Research Institute for Endocrine Sciences, Shahid Beheshti University of Medical Sciences, Tehran, Iran

**Keywords:** thyroid-stimulating hormone, free thyroxine, type 2 diabetes, joint modeling, incidence

## Abstract

**Background:**

Type 2 diabetes mellitus (T2DM) poses a significant public health challenge, contributing to considerable morbidity and mortality worldwide, which necessitates urgent preventive measures. Thyroid disorders, prevalent in many individuals, are intricately linked to metabolic health, yet studies on their relationship with T2DM yield inconsistent results—some suggesting an increased risk with abnormal thyroid hormone levels, while others indicate potential protective effects. This study investigated the association between changes in serum thyroid-stimulating hormone (TSH) and free thyroxine (FT4) levels and the incidence of type 2 diabetes mellitus.

**Methods:**

Data from 1938 individuals aged ≥20 in the Tehran Thyroid Study cohort were used, spanning four examination cycles from 1999 to 2012, with three-year intervals. TSH and FT4 levels were log-transformed and modeled as time-varying exposures to study their association with incident T2DM.

**Results:**

During a median follow-up of 9.43 years, 135 new T2DM cases were identified. The multivariable-adjusted joint model (JM) revealed that each unit increase in log-transformed TSH level was associated with a 25% decrease in T2DM incidence [HRs (95% CI): 0.75 (0.64-0.90)]. Conversely, each unit increase in FT4 level showed a marginally significant higher risk [1.06 (0.99-1.13); p-value=0.06].

**Conclusion:**

The findings of this study suggest that dynamic changes in serum thyroid hormones are associated with the development of T2DM. Rising TSH and decreasing FT4 over time are associated with a lower risk of diabetes. These findings suggest a complex interplay between thyroid function and the risk of T2DM, emphasizing the importance of monitoring thyroid hormone levels as a part of T2DM prevention strategies.

## Introduction

The increasing burden of T2DM is a substantial concern for healthcare systems worldwide, and it is associated with a range of acute and chronic complications, encompassing micro and macrovascular comorbidities such as cardiovascular, renal, neurological, infectious, and ophthalmic issues (1, 2). According to the 2023 report of the International Diabetes Federation (IDF), approximately 573 million individuals worldwide have T2DM, which is estimated to increase to 783 million cases by 2045 (2). The Middle East and North African region have the first rank worldwide; in Iran, a country in this region, incident diabetes has an incidence rate of 1% per year (3).

Several risk factors contribute to T2DM, including age, hypertension, family history of diabetes (FHD), obesity, and sociodemographic factors (4–7); Additionally, the association between thyroid dysfunction and T2DM has also been discussed (8). The effects of hypo- and hyperthyroid dysfunctions and even thyroid hormones within the reference range and T2DM have been reported with controversial results (9, 10). The underlying mechanisms and potential interactive stimuli are complex (9). Thyroid hormones unequivocally regulate glucose homeostasis through diverse mechanisms, including cellular glucose transport, intestinal glucose absorption, hepatic glucose secretion, and the secretion of insulin and counter-regulatory hormones (11). In the Rotterdam study, each unit increase in the thyroid-stimulating hormone (TSH) level increased the risk of incidence of T2DM by 9% in euthyroid individuals (9); however, in the Health in Pomerania study, each unit increase of serum TSH decreased the risk of incidence of T2DM by 6% (12). A meta-analysis suggested that higher serum TSH or free thyroxine (FT4) levels might increase the risk of T2DM. However, the increase was not statistically significant (13). Most of the studies conducted so far have employed logistic and Cox regression models, which typically consider only baseline thyroid function measurements when examining the association between thyroid hormones and the incidence of T2DM. However, these approaches overlook the dynamic nature of thyroid function over time. In contrast, our study addresses this gap by using JM that allow for individual-specific predictions that account for the variability in thyroid function trajectories over time. The JM provides more accurate and efficient parameters, reduces bias, and improves accuracy compared to previous models. This comprehensive approach provides more reliable estimates of the association between thyroid function and the risk of T2DM. Therefore, in the current study, we aimed to explore the dynamic association between longitudinal changes in serum TSH and FT4 and the incidence of T2DM during ten years of follow-up using JM of longitudinal and survival data among the Tehranian population.

## Materials and methods

### Study population

This study was conducted within the framework of the Tehran Thyroid Study (TTS), a prospective population-based cohort study of the residents of District 13 of Tehran from December 1999 to 2002. The first re-examination was performed between 2002 and 2005, followed by the second re-examination in 2005-2009 and the third re-examination in 2009-2012. The design and methodology of the TTS have been previously reported (14, 15). A total of 5769 adults aged ≥ 20 years were selected using a random sampling method within the Tehran Lipid and Glucose Study (TLGS) framework.

**Figure 1:**
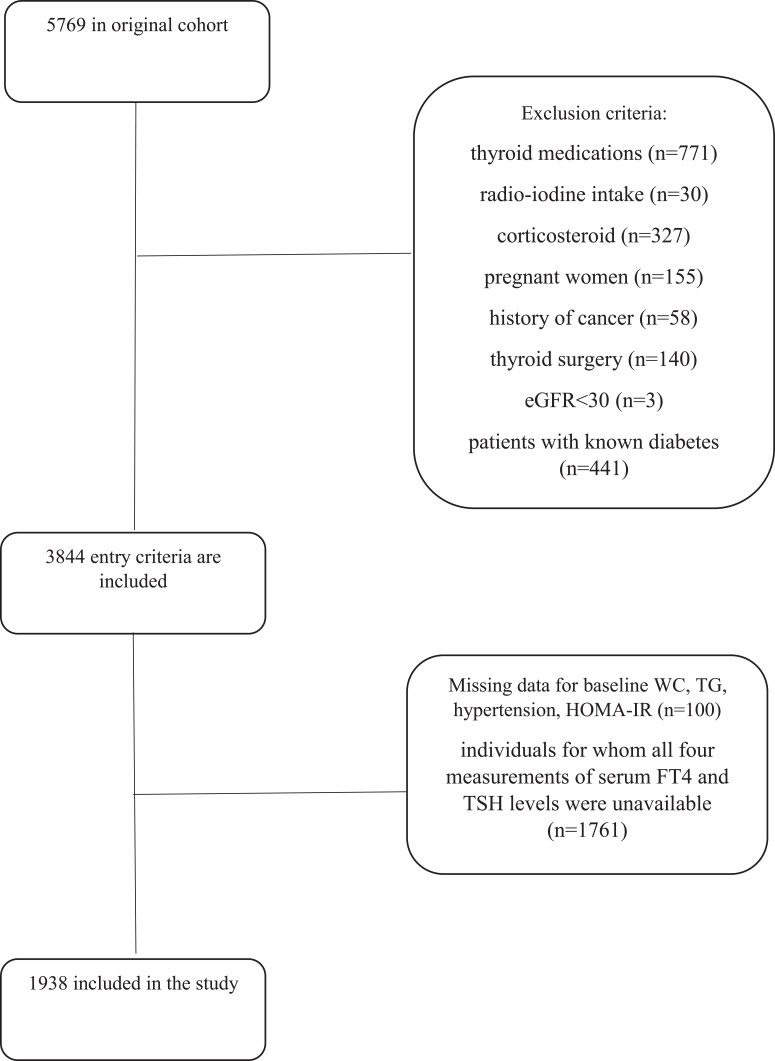
The flow of the participants through the study.

For the current study, adults aged ≥20 who had participated in the TTS from 1999 to 2002 (n=5769) were included. Individuals on thyroid medications (n=771), history of radio-iodine treatment (n=30), and corticosteroid (n=327) were excluded. We also excluded pregnant women (n=155), patients with known diabetes (n=441), those with a history of cancer (n=58), thyroid surgery (n=140), and eGFR<30 mL/min/1.73m^2^ (n=3). After further excluding those with incomplete data on baseline waist circumference, triglycerides, blood pressure, and Homeostatic Model Assessment of Insulin Resistance (n=100), as well as missing data on four measurements of serum FT4 and TSH levels (n=1761),1938 individuals (60.1% women) were included in the study (**Figure 1**).

### Clinical and laboratory measurements

Demographic information, medical history, and smoking habits were gathered from participants during interviews using a pretested questionnaire at baseline and follow-up assessment. Anthropometric measurements were obtained with participants wearing light clothing and after removing their shoes. Height and weight were assessed according to established standard procedures. WC was assessed at the umbilical level (16).

Blood pressure was assessed using a standardized mercury sphygmomanometer calibrated by the Iranian Institute of Standards and Industrial Research. It was measured twice on the right arm with the participant in a seated position following at least 15 minutes of rest. The average of these two measurements was considered the participant’s blood pressure.

Blood samples were gathered from individuals between 7-9 AM, following a 12- to 14-hour fasting period, while the subjects were in a seated position. These samples were then subjected to centrifugation for 30-45 minutes to separate the serum. All analyses were carried out on the same day as the blood sampling at the Research Institute for Endocrine Science laboratory. In addition to obtaining fasting blood samples for glucose measurement, a second blood sample was taken 120 minutes after glucose consumption to assess postprandial blood glucose levels. Blood glucose levels were measured using the Pars Azmoon kit and a colorimetric method with glucose oxidase. The intra- and inter-assay coefficients of variation (CV) of FPG and 2hPGC at baseline and follow-up phases were less than 2.3% (17). Serum FT4 and TSH levels were determined using the electrochemiluminescence immunoassay (ECLIA) method with the Roche Diagnostics kit. The analysis was performed using Germany’s Roche/Hitachi Cobas e-411 analyzer. Intra Interassay and intra-CVs were 4.5% and 1.5% for TSH, 3.7% and 1.3% for FT4 determinations, respectively. Thyroid peroxidase antibody (TPOAb) was measured using an immunoenzymometric assay (IEMA) with the Monobind kit (Costa Mesa, CA, USA) and the Sunrise ELISA reader (Tecan Co., Salzburg, Austria). The inter- and intra-assay CVs were 4.7% and 3.9%, respectively (15).

### Variable definitions

Body mass index (BMI) was computed by dividing weight in kilograms by the square of height in meters (m²). The definition of high blood pressure includes a DBP>= 90 mmHg, an SBP>= 140 mmHg, or the use of antihypertensive medication. A current smoker is described as an individual who smokes cigarettes either daily or occasionally. T2DM was confirmed among participants who had FPG ≥ 126 mg/dl or 2h-PGC ≥ 200 mg/dl or using anti-diabetic medication. Family history of diabetes (FH-DM) was defined as diabetes in first-degree relatives. HOMA-IR was calculated as fasting serum insulin (μIU/mL)×FPG (mg/dl)/405. Euthyroidism was specified as a FT4 of 0.91–1.55 ng/dL and a normal TSH within the 0.32–5.06 mIU/L range. Subclinical hypothyroidism was defined as TSH levels greater than 5.06, with FT4 levels between 0.91 and 1.55 ng/dl. Overt hypothyroidism was characterized by TSH levels greater than 5.06 and FT4 levels below 0.91. Subclinical hyperthyroidism was identified by TSH levels below 0.32 with FT4 levels between 0.91 and 1.55, and overt hyperthyroidism was defined by TSH levels below 0.32 and FT4 levels above 1.55 (18, 19).

### Statistics analysis

The baseline characteristics of the study population were presented using the mean (standard deviation: SD) for continuous variables and the number (%) for categorical variables. The median (interquartile range: IQR) was provided for covariates, demonstrating a skewed distribution.

The event date for incident T2DM was defined as the midpoint between the date of the follow-up visit when diabetes was initially detected and the date of the most recent preceding follow-up visit before the diagnosis (3, 20). For individuals who did not experience incident T2DM (i.e., censored individuals), the last follow-up time was considered their final examination date. The crude incidence rate (95% CI) of T2DM was calculated by dividing the number of new cases of T2DM by the number of person-years at risk. FT4 and TSH hormones were two repeated measurements that were used as time-dependent covariates in the development of the dynamic association model. In addition to the longitudinal measurements, fixed variables were selected based on a literature review of well-known covariates for T2DM, including age, gender, FHDM, BMI, hypertension, TG, and HOMA-IR (3).In the current study, the dynamic association model was fitted using JM of longitudinal and time-to-event data (longitudinal sub-model and survival sub-model) within a Bayesian framework, employing Markov Chain Monte Carlo (MCMC) methods (15000 Iteration) through Gibbs sampling by the JMbayes2 package version 0.4-5. A linear mixed-effects model was considered to evaluate the changes in longitudinal measurements of FT4 and the logarithmic transformation of TSH (to correct the skewed distribution) over time (**Supplementary Table S1**). Moreover, gender, TPOAb(IU/ml), and current smoking status were adjusted in the longitudinal part of the joint model. The following shows the related formula considering a linear mixed model for TSH as an example:


$$\begin{array}{c} m_{i}\left(t\right))=Log\;TSH_{i}\left(t\right)=\left(\beta_{0}+b_{0i}\right)+\beta_{1}+b_{1i})\times Time_{i}+\beta_{2}\times \\ \;gender+\beta_{3}\times current\;smoker+\\ \;\beta_{4}\times follow-up\;Log\;TPOAb+\epsilon_{i}\left(t\right) \end{array}$$

In the survival part of the model, the Cox regression was considered (**Supplementary Materials 1.2**). Two models were considered; model 1: baseline measurements of age, gender, FHD, BMI, hypertension, and TG, and Model 2: further adjustment for HOMA-IR as presented below:


$$\begin{array}{c} incidence\;DM=h_{0}\left(t\right)\;exp(\beta_{1}\;baseline\;Age+\beta_{2}\;Gender\left(Female\right)\\ \;+\beta_{3}\;baseline\;FHD+\\ \;\beta_{4}\;baseline\;BMI+\beta_{5}\;baseline\;hypertension+\beta_{6}\;baseline\;TG\\ \;+\beta_{7}\;basline\;HOMA-IR) \end{array}$$

The final JM serves as follows:


$$\begin{array}{c} incidence\;DM=h_{0}\left(t\right)\;exp(\beta_{1}\;baseline\;Age+\beta_{2}\;Gender\left(Female\right)+\\ \;\beta_{3}\;baseline\;FHD+\beta_{4}\;baseline\;BMI+\beta_{5}\;baseline\;hypertension+\\ \;\beta_{6}\;baseline\;TG+\beta_{7}\;basline\;HOMA-IR+\alpha_{1}m_{i}\left(t\right)) \end{array}$$

The parameter α measures the association between pre-selected features of the longitudinal process (log TSH) and the hazard for the incident T2DM.

The ‘best-fitted’ JM was selected by minimizing the deviance information criterion (DIC) (**Supplementary Table S2**). In addition to considering FT4 and TSH independently, we have also collapsed these covariates into a single JM. More explanations on model development, model selection, and model strategies are presented in **Supplementary Material 2**.

A sensitivity analysis was performed in euthyroid subjects to mitigate potential confounding effects from patients with abnormal thyroid levels, ensure the results focused on the impact of normal thyroid levels on the outcomes of interest, and enhance result validity, reliability, and accuracy. Patients with basal TSH levels below 0.3 mU/L or above 5.06 mU/L and FT4 levels below 0.91 ng/dl or above 1.55 ng/dl were excluded from the analysis.

The interaction effect of gender (p=0.08) and BMI (p=0.14) with time was not statistically significant; therefore, these two variables did not stratify the results.

Statistical analysis was done using R statistical software (version 4.3.0), and p-value ≤0.05 was considered statistically significant.

## Results

### Baseline characteristics

Totally 1938 participants (1165 women) with a mean ± SD age of 42.06 ± 13.06 years who were initially free of T2DM, were included.

**Table 1** presents the participants’ baseline characteristics. 26.7% of the participants had a family history of diabetes, 44.9% had a history of hypertension, and 9.4% reported a history of smoking. The mean serum FT4 level among the participants was 1.21 ± 0.24 ng/dL, and the median serum TSH level was 1.49 (0.92-2.41) mU/L. The baseline mean FPG and 2h-PGC levels were 89.48 ± 9.11 mg/dl and 104.21 ± 26.70 mg/dl, respectively. The baseline mean HOMA-IR was 1.80 ± 1.05.

**Table 1:** Baseline characteristics of included participants.

Total	1938(100)
**Female,n(%)**	1165 (60.1)
**FH-DM,n(%)**	517 (26.7)
**Current smoker, n(%)**	183 (9.4)
**Hypertension,n(%)**	871 (44.9)
**Overt hypothyroid**	20 (1.0)
**Subclinical hypothyroid**	71 (3.7)
**Overt hyperthyroid**	27 (1.4)
**Subclinical hyperthyroid**	71 (3.7)
**Age (year)**	42.06 ± 13.05
**BMI (kg/m^2^)**	26.89 ± 4.33
**FPG (mg/dl)**	89.48 ± 9.11
**2h-PGC (mg/dl)**	104.21 ± 26.70
**HOMA-IR**	1.80 ± 1.05
**Triglycerides (mg/dl)**	139.0 (94.0-198.0)
**TPOAb (IU/ml)**	4.81 (2.95-10.31)
**TSH (mU/L)**	1.49 (0.92-2.41)
**FT4 (ng/dl)**	1.21 ± 0.24

Values are shown as mean ± SD for continuous and number (%) for categorical variables, and TG, TPOAb, and TSH median (IQR).

SD, standard deviation; IQR, interquartile range; FH-DM, family history of diabetes; BMI, body mass index; FPG, fasting plasma glucose; 2h-PGC, 2-hour post–glucose challenge, HOMA-IR, Homeostatic model assessment of insulin resistance; TG, triglycerides; TSH, thyroid stimulating hormone; FT4, Free thyroxine.

Bold values indicate statistically significant results.

**Figure 2:**
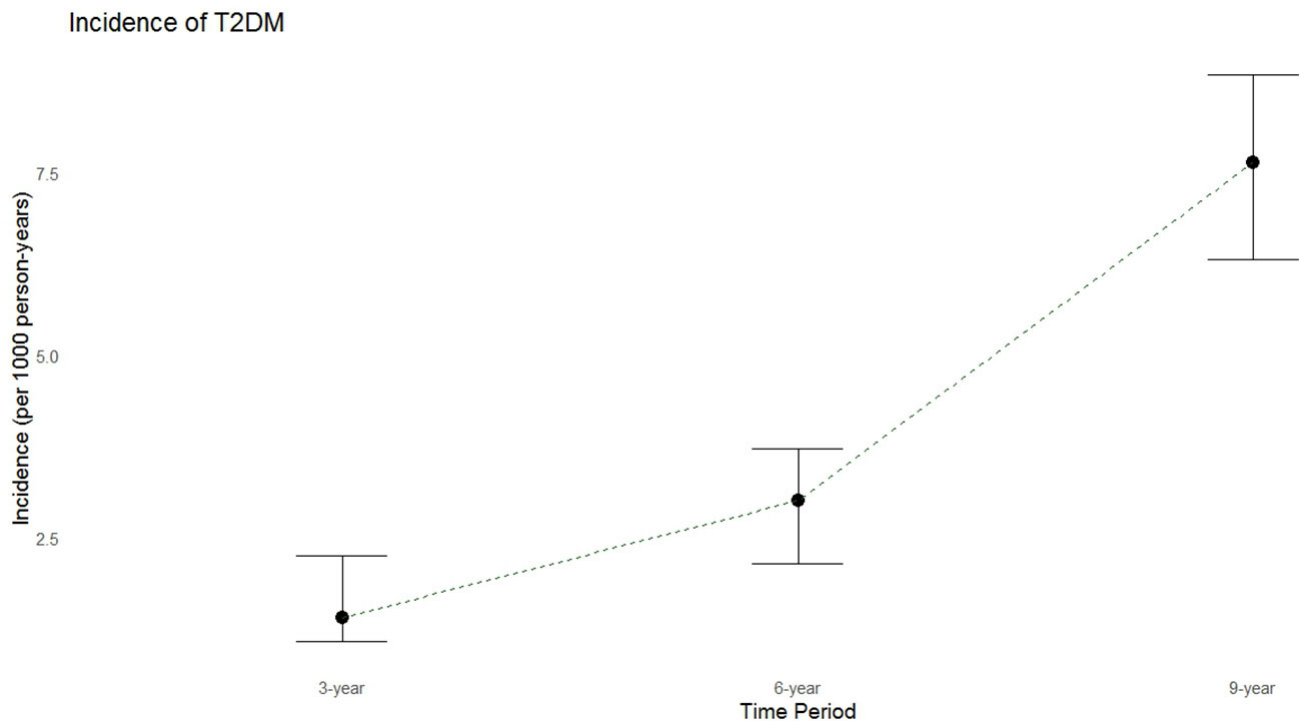
Incidence Rates of T2DM at 3, 6, and 9 Years Follow-Up of the TTS.

During a median follow-up period of 9.43 years (IQR: 6.4-10.4 years), 135 new cases of T2DM were identified. The crude annual incidence rate was 7.65 per 1000 person-years. The incidence rates of diabetes over 3 and 6 years were 1.4 and 3.0 per 1000 person-years, respectively (**Figure 2**).

### JM

**Figure 3:**
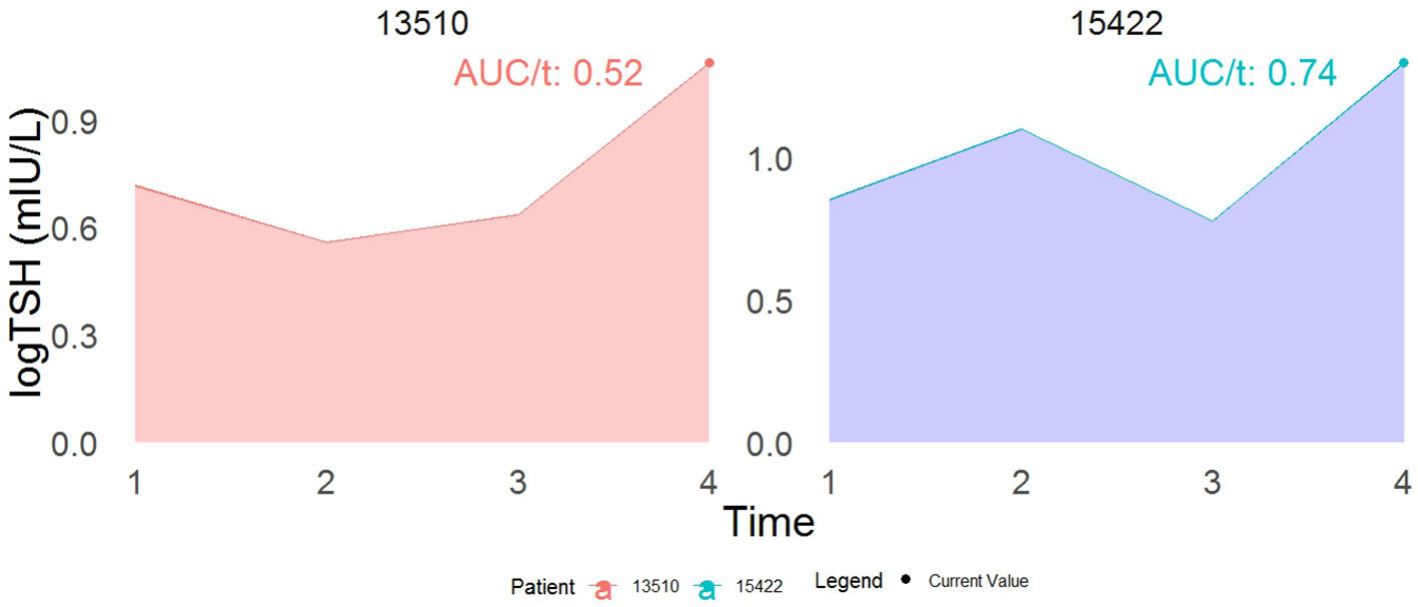
This example demonstrates the interpretation of a joint model incorporating both the current value (represented by the dot) and the cumulative area as association structures. While both patients show identical current values in log TSH, their cumulative area values differ. Specifically, patient 15422 has a 22% reduced risk of developing T2DM compared to patient 13510 at time t. The term AUC stands for the area under the curve.

**Table 2** shows that a higher TSH level (0.53-unit increase in the area under the longitudinal profile of logarithmic TSH equivalent to approximately a 1.7-fold increase in actual TSH levels is strongly associated with a significant decrease in incident T2DM. The joint model shows that each unit increase in serum logarithmic TSH levels () corresponds to a 22% (HR:0.78, 95%CI:0.66-0.95,p=0.01) and 25% (HR: 0.75,95%CI:0.64-0.90,p=0.003) decrease in the incidence of T2DM in model 1 (adjusted for gender, age, family history of diabetes, BMI, hypertension, and TG); and after further adjustment for HOMA-IR, respectively. **Figure 3** displays the total area under the longitudinal profile of logarithmic TSH for two patients.

**Table 2:** Joint analysis parameter estimated (95% confidence intervals) of longitudinal TSH hormone and time to incident T2DM: Tehran Thyroid Study 1999-2012.

	Model 1		Model 2	
	Estimate (95%CI)	p-value	Estimate (95%CI)	p-value
Longitudinal sub-model				
Fixed Intercept	-0.14 (0.03)	<0.001	-0.14 (0.03)	<0.001
Fixed slope: time	0.04 (0.001)	<0.001	0.04 (0.001)	<0.001
Log TPOAb, IU/ml	0.16 (0.002)	<0.001	0.16 (0.002)	<0.001
Gender, Female	0.24 (0.03)	<0.001	0.24 (0.03)	<0.001
Current Smoker, Yes	-0.10 (0.04)	0.02	-0.10 (0.04)	0.02
Survival sub-model				
	HR (95%CI)	p-value	HR (95%CI)	p-value
Gender, Female	1.09 (0.65-1.84)	0.75	1.08 (0.66-1.84)	0.76
Baseline Age, years	1.03 (1.02-1.05)	<0.001	1.04 (1.02-1.05)	<0.001
FHD, Yes	2.01 (1.42-2.86)	0.001	1.99 (1.40-2.83)	0.002
Baseline hypertension, Yes	1.18 (0.83-1.73)	0.37	1.11 (0.78-1.62)	0.58
Baseline TG, mg/dl	1.001 (1.0001-1.003)	0.04	1.001 (0.99-1.002)	0.15
BMI, kg/m^2^	1.09 (1.05-1.15)	<0.001	1.05 (1.01-1.09)	0.02
Baseline HOMA-IR	——————	——	1.38 (1.24-1.52)	<0.001
Joint model				
***α*_1_**	0.78 (0.66-0.95)	0.01	0.75 (0.64-0.90)	0.003

HR, hazard ratio; CI, confidence intervals; FHD, family history of diabetes; TG, triglycerides; BMI, body mass index, HOMA-IR, Homeostatic model assessment of insulin resistance; TSH, thyroid stimulating hormone; TPOAb, thyroid peroxidase antibody.

Model 1, adjusted for Gender, age, FHD, BMI, hypertension, and TG;

Model 2, Model 1+ HOMA-IR.

*α*_1_, Coefficient for longitudinal sub-model of Log TSH.

Bold values indicate statistically significant results.

**Figure 4:**
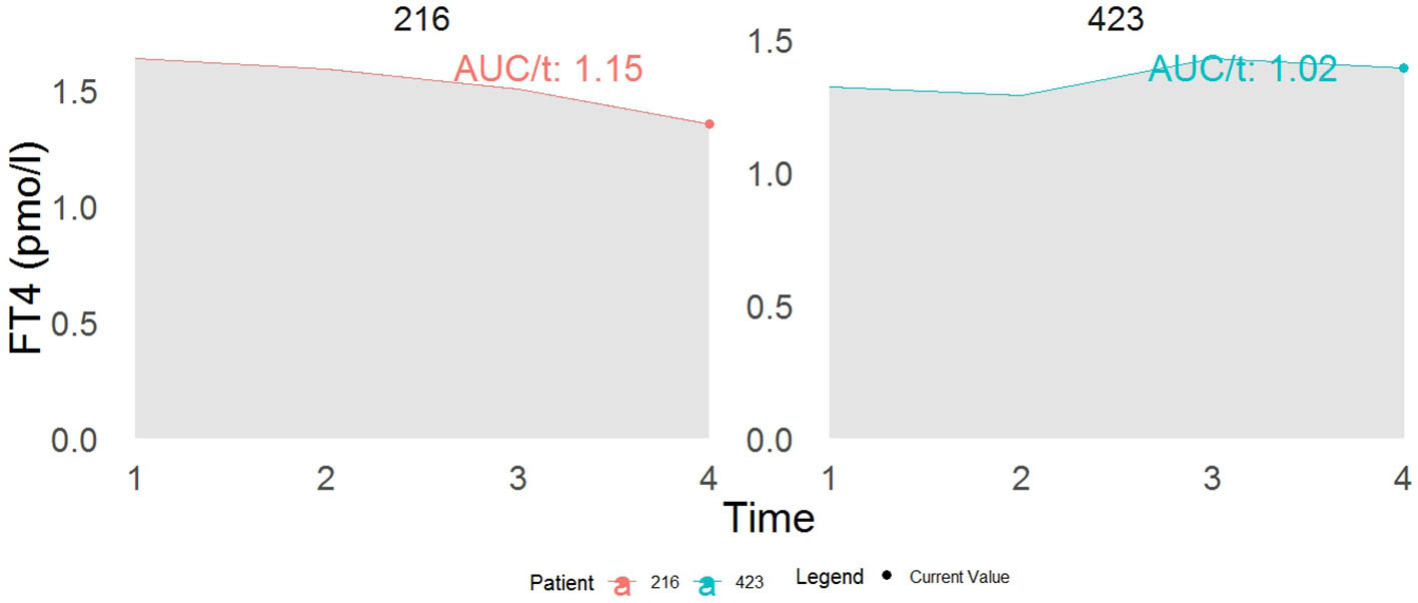
This example demonstrates the interpretation of a joint model incorporating both the current value (represented by the dot) and the cumulative area as association structures. While both patients show identical current values in FT4, their cumulative area values differ. Specifically, patient 216 has a 13% increased risk of developing T2DM compared to patient 423 at time t. The term AUC stands for the area under the curve.

The result from **Table 3** showed that a one-unit increase in serum FT4 level was associated with a marginally significant higher risk of diabetes (HR model 1:1.06, 95% CI: 0.99–1.11, p = 0.07; HR model 2: 1.06, 95% CI: 0.99–1.13, p = 0.06). **Figure 4** displays the total area under the longitudinal profile of FT4 for two patients.

**Table 3:** Joint analysis parameter estimated (95% confidence intervals) of longitudinal FT4 hormone and time to incident T2DM: Tehran Thyroid Study 1999-2012.

	Model 1		Model 2	
	Estimate (95%CI)	p-value	Estimate (95%CI)	p-value
Longitudinal sub-model				
Fixed Intercept	16.37 (0.10)	<0.001	16.37 (0.10)	<0.001
Fixed slope: time	-0.07 (0.01)	<0.001	-0.07 (0.01)	<0.001
Log TPOAb, IU/ml	-0.18 (0.03)	<0.001	-0.18 (0.03)	<0.001
Gender, Female	-0.86 (0.09)	<0.001	-0.86 (0.09)	<0.001
Current Smoker, Yes	0.34 (0.12)	0.004	0.34 (0.12)	0.004
Survival sub-model				
Covariate	HR (95%CI)	p-value	HR (95%CI)	p-value
Gender, Female	1.07 (0.63-1.80)	0.80	1.07 (0.65-1.80)	0.79
Baseline Age, years	1.03 (1.02-1.05)	<0.001	1.04 (1.02-1.05)	<0.001
FHD, Yes	2.03 (1.42-2.86)	<0.001	1.99 (1.40-2.83)	0.002
Baseline hypertension, Yes	1.17 (0.82-1.69)	0.40	1.11 (0.77-1.66)	0.57
Baseline TG, mg/dl	1.001 (1.0002-1.003)	0.03	1.001 (0.99-1.002)	0.11
BMI, kg/m^2^	1.09 (1.05-1.14)	<0.001	1.05 (1.01-1.09)	0.02
Baseline HOMA-IR	————–	———–	1.36 (1.22-1.51)	<0.001
Joint model				
***α*_2_**	1.06 (0.99-1.11)	0.07	1.06 (0.99-1.13)	0.06

HR, hazard ratio; CI, confidence intervals; FHD, Family History of Diabetes; TG, triglycerides, BMI, body mass index, HOMA-IR, Homeostatic model assessment of insulin resistance; FT4, Free thyroxine; TPOAb, thyroid peroxidase antibod;

Model 1, adjusted for Gender, age, FHD, BMI, hypertension, and TG;

Model 2, Model 1+ HOMA-IR.

*α*_2_, Coefficient for longitudinal sub-model of FT4.

Bold values indicate statistically significant results.

As the correlation between serum FT4 and TSH levels was not strong across all four study phases (R-squared < 0.20, **Supplementary Figure S1**), we further investigated the results using a multivariable joint model (with both FT4 and TSH) (**Table 4**). A one-unit increase in serum logarithmic TSH levels was associated with a lower risk of developing T2DM (HR: 0.78, 95% CI: 0.64-0.97), independent of FT4 levels; however, FT4 was not associated with the risk of diabetes (**Table 4**).

**Table 4:** Multivariate joint analysis parameter estimated (95% confidence intervals) of longitudinal TSH and FT4 hormone with time to incident T2DM: Tehran Thyroid Study 1999-2012.

	Model 1		Model 2	
	Estimate (95%CI)	p-value	Estimate (95%CI)	p-value
Longitudinal sub-model				
Fixed Intercept	-0.14 (0.03)	<0.001	-0.14 (0.03)	<0.001
Fixed slope: time	0.04 (0.002)	<0.001	0.04 (0.002)	<0.001
Log TPOAb, IU/ml	0.16 (0.01)	<0.001	0.16 (0.01)	<0.001
Gender, Female	0.24 (0.03)	<0.001	0.24 (0.03)	<0.001
Current Smoker, Yes	-0.09 (0.04)	0.03	-0.09 (0.04)	0.03
Survival sub-model				
	HR (95%CI)	p-value	HR (95%CI)	p-value
Gender, Female	1.09 (0.66-1.85)	0.73	1.09 (0.66-1.88)	0.73
Baseline Age, years	1.03 (1.01-1.05)	<0.001	1.04 (1.02-1.05)	<0.001
FH-DM, Yes	2.03 (1.43-2.85)	<0.001	2.01 (1.42-2.86)	<0.001
Baseline hypertension, Yes	1.17 (0.81-1.72)	0.38	1.10 (0.76-1.63)	0.60
Baseline TG, mg/dl	1.001 (1.0001-1.003)	0.03	1.001 (0.99-1.002)	0.13
BMI, kg/m^2^	1.09 (1.05-1.14)	<0.001	1.05 (1.01-1.09)	0.02
Baseline HOMA-IR	——————	——	1.38 (1.25-1.52)	<0.001
Joint model				
***α*_1_**	0.81 (0.66-1.00)	0.05	0.78 (0.64-0.97)	0.02
***α*_2_**	1.02 (0.95-1.09)	0.48	1.02 (0.94-1.09)	0.57

HR, hazard ratio; CI, confidence intervals; FH-DM, family history of diabetes; TG, triglycerides; BMI, body mass index; HOMA-IR, Homeostatic model assessment of insulin resistance;TSH, thyroid stimulating hormone; TPOAb, thyroid peroxidase antibody.

Model 1, adjusted for Gender, age, FHD, BMI, hypertension, and TG;

Model 2, Model 1+ HOMA-IR.

α_1_, Coefficient for longitudinal sub-model of Log TSH.

α_2_, Coefficient for longitudinal sub-model of FT4.

Bold values indicate statistically significant results.

### Sensitivity analysis

A sensitivity analysis was conducted on euthyroid subjects to see whether the association exists in euthyroid individuals; and the results indicated that the parameter estimates remain constant in the euthyroid group (**Supplementary Table S3**).

As another sensitivity analysis, adjustments were made for changes in BMI over time, and the results remained unchanged.

## Discussion

In this population-based TTS, we used a JM approach to evaluate the association between longitudinal serum TSH and FT4 levels and the risk of T2DM. Notably, no prior study has utilized joint models to investigate the association between thyroid hormones and the incidence of T2DM, making our approach novel in this context. We found a significant reverse association between serum TSH levels and the risk of T2DM. Each unit increase in serum logarithmic TSH levels corresponded to a 25% decrease in the incidence of T2DM Although each unit increase in serum FT4 was associated with a 6% increased risk of T2DM; the multivariable JM, using both FT4 and TSH, indicated a significant association only for TSH. Therefore, the trend of serum TSH levels over time might be associated with the development of T2DM.

Given the impact of thyroid hormones on lipids, insulin secretion, and carbohydrate metabolism, it is hypothesized that thyroid hormones could influence the development of T2DM (21, 22). Thyroid hormones have been found to stimulate the basal expressions of glucose transporters, which play a crucial role in regulating intracellular glucose uptake on the surface of myocytes (23). Various studies have reported an association between thyroid dysfunction and type 2 diabetes (9, 12). In a meta-analysis, hypothyroidism was shown to be associated with an increased risk of T2DM; however, hyperthyroidism and the normal range of thyroid hormones were not significantly related to the risk of diabetes (24). Thyroid dysfunctions increase insulin resistance in muscle and adipose tissue while decreasing glucose transport in myocytes (25, 26). Hypothyroidism can lead to insulin resistance, possibly due to changes in GLUT4 translocation, the effects of leptin, and an increase in free fatty acids (25). This study found that decreasing serum TSH and increasing FT4 levels over time were associated with developing T2DM, which favors hyperthyroidism. The serum FT4 level exhibited marginal significance, correlating with a 6% risk of T2DM. However, this significance was inconclusive due to the study’s insufficient sample size and power. Indeed, with a larger sample size, this association is expected to achieve significance, potentially increasing the risk.

Hyperthyroidism can cause hyperglycemia by increasing GLUT2 transporters in the liver, promoting lipolysis and nonoxidative glucose disposal, and increasing hepatic glucose output. Additionally, hyperthyroidism is associated with increased glucose use in skeletal muscle and reduced glycogen synthesis, which may result in a hypoglycemic state, counteracting the pathways that lead to hyperglycemia (27, 28). These findings highlight the complex interplay between thyroid function and glucose metabolism and suggest that maintaining optimal thyroid function may be necessary for overall metabolic health (10).

In contrast with our results, the study conducted by Chakar et al (9). in 2016, 8452 euthyroid individuals aged over 55 with a follow-up period of 7.9 years found that a one-unit increase in serum TSH level (HR: 1.09, 95%CI: 1.06-1.12), and a one unit decrease in serum FT4 (HR:1.06, 95%CI:1.02-1.10) were associated with the increased risk of T2DM; however, the study included elderly participants, and TSH was measured only at baseline which did not consider the effect of changes in TSH levels overtime on the development of T2DM. Similar to the current study, the study by Ittermann et al. (12) on 6206 individuals aged 30-60 y. in Denmark showed that a one-unit increase in serum TSH (HR: 0.84, 95%CI: 0.66-1.08) and decrease in serum FT4 (HR:0.97, 95%CI: 0.93-1.0) levels reduced the risk of T2DM, but the association was not statistically significant. The two studies mentioned above utilized Cox regression models that solely considered baseline thyroid hormone levels, neglecting the dynamic changes in thyroid hormones over time. In a meta-analysis conducted on 21,080 individuals, each unit increase in both serum FT4 and TSH levels increased the risk of T2DM, but the associations were not statistically significant (24); the non-significant association was attributed to the J- shaped or inverted J-shaped association between thyroid hormones and T2DM. Bos et al. (29) identified a connection between genetic variants associated with thyroid metabolism and insulin resistance; this observation implies that thyroid dysfunction may contribute to the development of T2DM. In their study, Bos and colleagues found some suggestive evidence, using median weighted estimator analysis, that there might be an association between a higher TSH level and a lower risk of T2DM. The estimated beta coefficient was -0.139, with a 95% confidence interval ranging from -0.301 to 0.023; however, this association was not statistically significant. When using inverse-variance weighted analyses to combine the effects of individual genetic instruments on the outcomes, there was no evidence of an association between TSH level and the risk of T2DM. The odds ratio was 0.91 per 1 standard deviation higher TSH, with a 95% confidence interval ranging from 0.78 to 1.07. A more recent Mendelian randomization study conducted by Kuś et al. (30) reported no discernible association between thyroid hormones and type 2 diabetes in their primary analyses; nevertheless, upon excluding pleiotropic instruments, Kuś et al. (30) noted a statistically significant association, indicating that higher levels of TSH were correlated with a reduced risk of T2DM (OR:0.95,95%CI: 0.91-0.98, p=0.0025); this suggests that within the normal range, higher TSH levels may be associated with a lower risk of T2DM like the current study. The presence of genes with pleiotropic effects could explain the observed discrepancy between the results of observational studies and Mendelian randomization analyses.

The significant association between serum TSH level and T2DM in the current study was observed after adjusting for age, gender, BMI, TG, hypertension, and FHD, and even after adjustment for HOMA-IR, suggesting that the association exists independent of HOMA-IR.

Also, the evidence suggests that the association between thyroid hormones and the occurrence of T2DM may be mediated by other factors, such as increased body mass index and impaired insulin secretion (27).

This study is strengthened by its longitudinal population-based design with a long follow-up period and serum TSH and FT4 values evaluation. The study is outstandingly powered by the joint model in which we investigated the effect of changes in serum TSH and FT4 levels on the incidence of T2DM. We chose the best model among the five joint models based on DIC criteria. The cumulative effect area structure joint model is more robust than other structures. Cox models underestimate the association of covariates with the outcome due to measurement error. In contrast, joint models have been shown to produce smaller standard errors, indicating greater efficiency compared to Cox models (31). A joint model, which combines longitudinal and time-to-event data, offers a valuable approach for assessing the impact of a longitudinal covariate, measured with error, on the time to the event of interest. JM produce more efficient and unbiased estimates of the association of longitudinal variables over time to events and reduce bias in the estimates. We analyzed survival and longitudinal changes in serum TSH and FT4 levels using the JM.

This study was limited to not having data regarding FT3 or T3 and FT4/FT3. In this study, HbA1C levels were not measured; however, the diagnosis of diabetes was based on the standard definition used in the TLGS; utilizing FPG and 2h-PGC glucose concentrations.

## Conclusion

Our study is the first to examine the association between serum thyroid hormone levels and the incidence of T2DM using the JM of longitudinal and time to event method using the whole longitudinal thyroid hormone profile of the patients over ten years. The longitudinal trend of serum thyroid hormones toward hyperthyroidism, especially the downward trend in serum TSH values would be associated with the development of T2DM. Understanding individual-specific TSH set points provides valuable insights into the function of the thyroid gland and helps guide diagnosis and treatment decisions. As the TSH set-point varies significantly among individuals and is not clinically applicable, monitoring the trend of serum thyroid hormones and their potential adverse outcomes over a long-term period might be helpful as an alternative approach in clinical settings. This helps physicians tailor their approach to treatment, ensuring that the patient’s thyroid hormone levels are optimized for their specific needs.

## Data Availability

The datasets presented in this study can be found in online repositories. The names of the repository/repositories and accession number(s) can be found in the article/**Supplementary Material**.
