# Supplementary material for: Association between changes in thyroid hormones and incident type 2 diabetes using joint models of longitudinal and time-to-event data: more than a decade follow up in the Tehran thyroid study
Source: Front Endocrinol (Lausanne). 2024 Dec 13;15:1475286. doi: 10.3389/fendo.2024.1475286 (PMC11671262; doi:10.3389/fendo.2024.1475286)
Supplement: Supplementary file 1 [file DataSheet1.docx]

[Supplementary documents 2](#_Toc173110638)

[1. Joint Model 2](#_Toc173110639)

[1.1 Mixed-effects modeling 2](#_Toc173110640)

[1.2 Survival model 4](#_Toc173110641)

[1.3 Joint model selection 5](#_Toc173110642)

[2 JM approaches 6](#_Toc173110643)

[2.1 Current value 6](#_Toc173110644)

[2.2 Time‑dependent slopes value 6](#_Toc173110645)

[2.3 Cumulative affect area value 7](#_Toc173110646)

[3 Multivariate joint model 8](#_Toc173110647)

[Table S1: Selection criteria for the best longitudinal structure 9](#_Toc173110648)

[Table S2: model fitness parameter among five different association structures of joint modeling models 9](#_Toc173110649)

[Table S3: Sensitivity analysis of a Joint Analysis parameter estimated (95% confidence intervals) of longitudinal LogTSH and FT4 hormone and time to incident T2DM in euthyroid individual: Tehran Thyroid Study 1999-2012 10](#_Toc173110650)

Figure S1: Scatter plot and Pearson correlation coefficient between TSH and FT4 at baseline and re-examination measurements………………………………………………….11

# Supplementary documents

# Joint Model

A joint model (JM), which combines longitudinal and time-to-event data, offers a valuable approach for assessing the impact of a longitudinal covariate, measured with error, on the time to the event of interest. Additionally, it provides a framework for evaluating the predictive capability of a biomarker on survival. A joint model comprises two primary components: a longitudinal component and a time-to-event (survival) component. The longitudinal component focuses on modeling the trajectory of the longitudinal outcome over time, while the time-to-event component is concerned with modeling the hazard or survival function for the event of interest. By simultaneously analyzing these two components, a joint model allows for a more comprehensive understanding of the relationship between the longitudinal covariate and the event outcome, while accounting for the inherent correlation between the two types of data.

## Mixed-effects modeling

In numerous research studies, particularly in the clinical field, it is common to encounter data that are collected longitudinally or in clusters. The presence of correlation between observations renders the use of fixed effects models inappropriate, as these models assume independence among the observations.

The linear mixed model (LMM) is widely employed as a random effects model in situations involving continuous repeated measurements from longitudinal responses. This model is applicable when the measurements are obtained from the same or related subjects at different time points, and in both scenarios, the responses are expected to exhibit correlation.

The mixed model incorporates both fixed and random parameters to provide an appropriate characterization of each individual's trend. The fixed effect coefficients are regarded as population constants and reflect the average trends across all individuals, similar to the coefficients estimated in a regression model. However, in a repeated measurement design, it is necessary to account for the variability that arises for each individual. This variability is captured by a set of random or latent effects.

In a simple model, our focus is on a longitudinally measured variable, yi(t), which is assumed to follow a normal distribution. To capture the relationship between the longitudinal variable and the associated covariates, we formulate a linear mixed-effects model ([1](#_ENREF_1)):

$$Y_{i}=X_{i}(t)\beta+Z_{i}{\left( t \right)b}_{i}+\varepsilon_{i}(t)$$

$$b_{i}\sim N(0.D)$$

$$\varepsilon_{i}(t)\sim N(0.\sigma_{\varepsilon}^{2})$$

Here, x_i_(t) represents the covariate vector for the fixed-effects coefficient vector β, while Z_i_(t) represents the covariate vector for the random-effects vector bi, for subject i (where i:1,…,n). The random effects, bi, are assumed to be mutually independent and multivariate normally distributed with a mean of zero and a variance-covariance matrix, D. The term ε_i_(t) represents the within-subject error, which is assumed to follow a normal distribution with a mean of zero and a constant variance, σ^2^ ([2](#_ENREF_2)).

In our study, the dependent variables of thyroid stimulating hormone(TSH) and free thyroxine (FT4) levels were measured at different time points with different individual characteristics. To model the longitudinal measurements of TSH and FT4 levels over time within an individual, a linear mixed-effects model was fitted, which can be defined by the following equation:

$$m_{i}\left( t \right)={Log TSH}_{i}(t)={(\beta}_{0}+b_{0i})+{(\beta}_{1}+b_{1i})\times{Time}_{i}+\beta_{2}\times gender+\beta_{3}\times follow-up current smoker+\beta_{4}\times follow-up Log TPOAb+\varepsilon_{i}\left( t \right)$$

$$m_{i}(t))={FT4}_{i}(t)={(\beta}_{0}+b_{0i})+{(\beta}_{1}+b_{1i})\times{Time}_{i}+\beta_{2}\times gender+\beta_{3}\times current smoker+\beta_{4}\times follow-up Log TPOAb+\varepsilon_{i}\left( t \right)$$

To determine the optimal time structure in the mixed-effects model, we assessed both linear and non-linear structures of second and third-order time components, selecting the best structure based on the lowest Bayesian Information Criterion (BIC) (Supplementary Table 1).

## Survival model

The Cox proportional hazards model is essentially a semi-parametric model that is commonly used in medical research to investigate the relationship between the survival time of patients and one or more predictor variables. The Cox proportional hazards (PH) model is based on the assumption that covariates have a multiplicative effect on the hazard for an event at time t for each subject i, where i:1,…,n. This can be expressed as

$$hi\left( t \right)=h_{0}\left( t \right)exp\{\gamma X_{i}\}$$

In the above equation, h_0_(t) represents the baseline hazard function, Xi is a 1×p vector of time-independent covariates for subject i, and γ is the corresponding p×1 vector of regression coefficients, with p denoting the number of parameters([3](#_ENREF_3)). The Cox PH model assumes that hazards are proportional between time-independent conditions, and the hazard ratio, exp(γ), is assumed to remain constant over time. In our study, we have identified several predictive factors for type 2 diabetes. According to Dr. Derakhshan et al.'s study([4](#_ENREF_4)), gender, age, waist circumference (WC), family history of diabetes (FHD), triglyceride (TG), hypertension, 2-hour post–glucose challenge (2h-PCG), and fasting plasma glucose (FPG) were associated with the onset of type 2 diabetes and were included as variables in the Cox regression model.

These baseline factors include age, gender, WC, FHD, TG, hypertension, FPG, and 2h-PCG. The model is structured as follows:

$$incidence DM=h_{0}(t) exp({\beta_{1} baseline Age+\beta_{2} Gender \left( Female \right)+\beta_{3} baseline FHD+\beta_{4} baseline BMI+\beta}_{5} baseline hypertension+\beta_{6} baseline TG+\beta_{7}basline HOMA-IR+\alpha_{i}m_{i}\left( t \right))$$

The parameter α measures the association between pre-selected features of the longitudinal process (TSH/FT4) and the hazard for the incident T2DM.

## Joint model selection

To fully specify the joint model, the true and unobserved value of the longitudinal covariate at time t, mi(t), which is estimated in the longitudinal model, is incorporated into the survival model. This linking of the two models creates a single, comprehensive joint model. By including the longitudinal covariate in the survival model, we can examine the impact of the covariate on the hazard of the event of interest while accounting for the correlation between the longitudinal and survival outcomes.

In the standard formulation of joint models, the relationship between the subject-specific marker and the risk of an event occurring at the same time t is typically modeled through an association parameter 𝛼. However, this functional form may not be sufficient to describe the association structure between outcomes in all settings. As a result, alternative association structures have been proposed in the literature. Two such structures are: (1) allowing the risk of an event to depend on the slope of the longitudinal profile, and (2) postulating that the risk of an event at time t is dependent on the integrated longitudinal profile, which represents a cumulative area effect. These alternative structures may be more appropriate in certain contexts and can provide a more accurate representation of the relationship between the subject-specific marker and the risk of an event.

$$m_{i}(t))={FT4}_{i}(t)={(\beta}_{0}+b_{0i})+{(\beta}_{1}+b_{1i})\times{Time}_{i}+\beta_{2}\times gender+\beta_{3}\times current smoker+\beta_{4}\times follow-up Log TPOAb+\varepsilon_{i}\left( t \right)$$

# JM approaches

## Current value

The first association structure between the longitudinal and time-to-event sub-models is commonly referred to as the "current value" approach. In this approach, the assumption is made that for an individual i, the true value yi(t) of the longitudinal measure at time t is predictive of the risk ℎi(t) of experiencing the event at the same time t. This means that the current underlying value of the subject-specific marker is directly associated with the risk of the event occurring at that specific time point. This can be expressed by the following formula:

$$hi\left( t \right)=h_{0}\left( t \right)exp\{\gamma^{T}X_{i}+\alpha m_{i}\left( t \right)\}$$

Where ho (t) is the baseline hazard function, the effect parameters γ describe how the hazard varies as a function of explanatory covariates Xi. The parameter α quantifies the association between a priori selected features of the longitudinal process and the hazard for the event at time t ([5](#_ENREF_5), [6](#_ENREF_6)). The model in our study is as follows:

$$hi\left( t \right)=h_{0}\left( t \right)exp\{\beta_{1}\times age+\beta_{2}\times gender+\beta_{3}\times FHD+\beta_{4}\times BMI+\beta_{5}\times TG+\beta_{6}\times hypertension+\beta_{7}\times HOMA-IR+\alpha_{1}m_{i}(t)\}$$

The α_1_ reflects the increase in the log hazard ratio of the event for a 1-unit increase in the longitudinal profile at the same time t.

## Time‑dependent slopes value

The current value parameter represents a specific time point and doesn't influence the rate of change. One path may be increasing while another is decreasing. This leads to the introduction of the slope values in the second part of the longitudinal survival model. This aspect accounts for the rate of change in measurements at time t, which is estimated as the derivative of m_i_(t)([5](#_ENREF_5), [6](#_ENREF_6)).

$$hi\left( t \right)=h_{0}\left( t \right)exp\{\gamma^{T}X_{i}+\alpha_{2} m_{i}^{'} (t) \}$$

where $m_{i}^{'} \left( t \right)=\frac{d}{dt}m_{i}(t)$.

The parameter α_2_ quantifies the association between the velocity of the true longitudinal trajectory at time t and the concurrent event risk, under the condition that mi(t) remains constant.

The model in our study is as follows:

$$hi\left( t \right)=h_{0}\left( t \right)\exp\left\{ \beta_{1}\times age+\beta_{2}\times gender+\beta_{3}\times FHD+\beta_{4}\times BMI+\beta_{5}\times TG+\beta_{6}\times hypertension+\beta_{7}\times HOMA-IR++\alpha_{2}\acute{m_{i}}\left( t \right) \right\}$$

That α_2_ represents the association between the slope of longitudinal changes in TSH or FT4 and the incidence of type 2 diabetes.

## Cumulative affect area value

This structure enables the complete trajectory of the longitudinal marker to be linked with the hazard for an event by incorporating the integral of the longitudinal trajectory into the linear predictor of the survival sub-model. The integral represents the cumulative effect of the longitudinal outcome up to time point t. This is expressed as:

$$hi\left( t \right)=h_{0}\left( t \right)exp\{\gamma^{T}X_{i}+\alpha_{3}\int_{t_{0}}^{t} m_{i}\left( s \right)ds \}$$

Here, α represents the degree of association between the risk for an event at time point t and the area under the longitudinal trajectory up to the same time t. The area under the longitudinal trajectory is considered a suitable summary of the entire trajectory. This parameterization has been demonstrated to enhance the statistical power of the analyses([5-7](#_ENREF_5)). The model in our study is as follows:

$$hi\left( t \right)=h_{0}\left( t \right)exp\{\beta_{1}\times age+\beta_{2}\times gender+\beta_{3}\times FHD+\beta_{4}\times BMI+\beta_{5}\times TG+\beta_{6}\times hypertension+\beta_{7}\times HOMA-IR++\alpha_{3}\int_{t_{0}}^{t} m_{i}\left( s \right)ds\}$$

The coefficient $\alpha_{3}$represents the association between the area under the longitudinal trajectory and the risk of type 2 diabetes. This coefficient indicates the extent to which changes in the longitudinal trajectory of a variable (Log TSH or FT4), are related to the risk of developing type 2 diabetes.

# Multivariate joint model

A multivariate joint model is a statistical approach that simultaneously analyzes multiple dependent variables within a single framework. This method is particularly useful when the variables are correlated or share common underlying factors. By considering the joint distribution of these variables, the model can capture complex relationships and dependencies, providing a more comprehensive understanding of the underlying processes. These models enable researchers to account for the correlations between variables, leading to more accurate predictions and a deeper insight into the underlying dynamics of the studied phenomena([8](#_ENREF_8), [9](#_ENREF_9)). The model in our study is as follows:

$$hi\left( t \right)=h_{0}\left( t \right)exp\{\beta_{1}\times age+\beta_{2}\times gender+\beta_{3}\times FHD+\beta_{4}\times BMI+\beta_{5}\times TG+\beta_{6}\times hypertension+\beta_{7}\times HOMA-IR+\alpha_{1}m_{i1}\left( t \right)+\alpha_{2}m_{i2}\left( t \right)\}$$

The α_1_ and α_2_ reflect the increase in the log hazard ratio of the event for a 1-unit increase in the longitudinal profile at the same time t for log TSH and FT4, respectively.

| Table S1: Selection criteria for the best longitudinal structure | | | | |
| --- | --- | --- | --- | --- |
| longitudinal structure | Log TSH | | FT4 | |
|  | AIC | BIC | AIC | BIC |
| Time | 15575.77 | 15925.57 | -5579.53 | -5651.54 |
| Time+ I(time^2) | 15607.37 | 15975.79 | -5644.83 | 5742.16 |
| Time+ I(time^2)+I(time^3) | 15622.13 | 16000.5 | -5561.10 | -5854.57 |
| AIC, Akaike information criteria; BIC, Bayesian information criteria | | | | |

| Table S2: model fitness parameter among five different association structures of joint modeling models | | |
| --- | --- | --- |
|  | DIC | DIC |
| Current value | 17307.11 | -4583.66 |
| Slope value | 17305.63 | -4592.28 |
| Current value plus slope | 17308.54 | -4593.26 |
| Cumulative effects (area) | 17302.37 | -4554.67 |
| Current value plus area | 17306.75 | -4598.82 |
| DIC, deviance information criterion | | |

| Table S3: Sensitivity analysis of a Joint Analysis parameter estimated (95% confidence intervals) of longitudinal LogTSH and FT4 hormone and time to incident T2DM in euthyroid individual: Tehran Thyroid Study 1999-2012 | | | | |
| --- | --- | --- | --- | --- |
|  | Model 1 | | Model 2 | |
| Joint model | HR (95%CI) | P-value | HR (95%CI) | P-value |
| $\boldsymbol{\alpha}_{\boldsymbol{1}}$ | 0.69 (0.54-0.96) | 0.04 | 0.71(0.56-0.94) | 0.009 |
| $\boldsymbol{\alpha}_{\boldsymbol{2}}$ | 1.05(0.92-1.18) | 0.07 | 1.06(0.93-1.20) | 0.07 |
| HR, hazard ratio; CI, confidence intervals;  Model 1, adjusted for Gender, age, FHD, BMI, hypertension, and TG;  Model 2:Model 1+ HOMA-IR  α_1_: Coefficient for longitudinal sub-model of Log TSH  α_2_: Coefficient for longitudinal sub-model of FT4 | | | | |


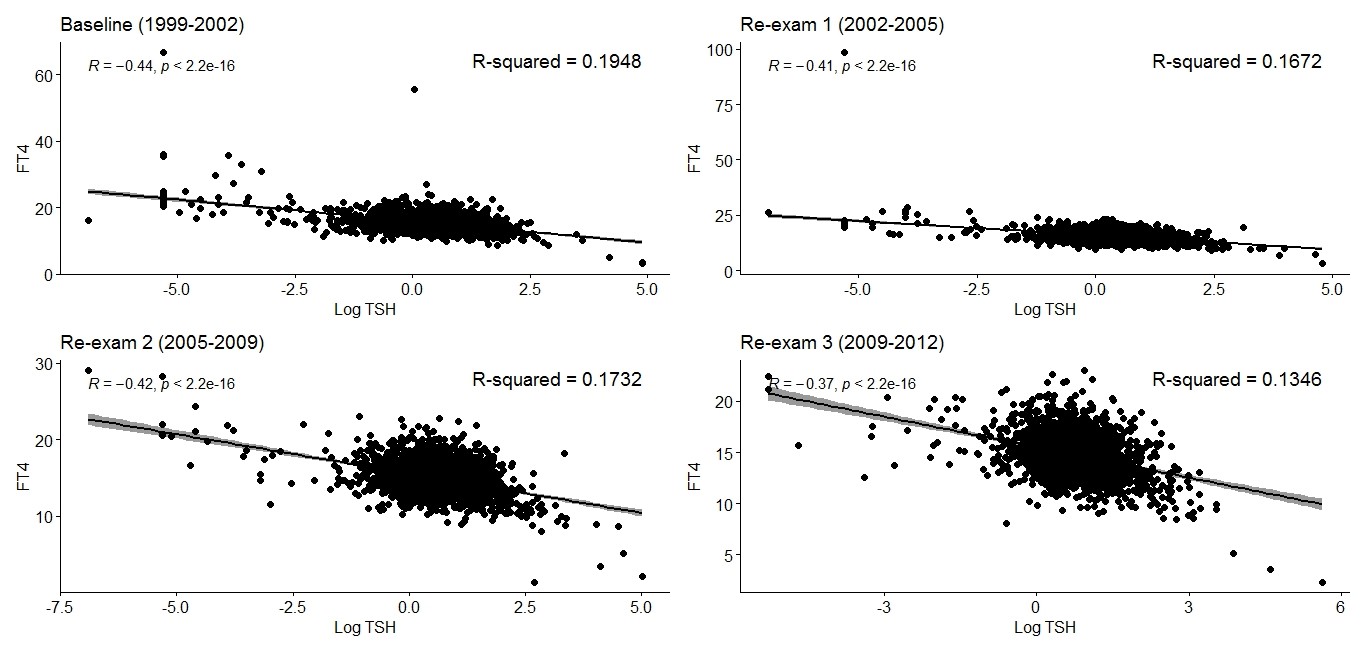


**Figure S1: Scatter plot and Pearson correlation coefficient between TSH and FT4 at baseline and re-examination measurements.**

TSH: thyroid stimulating hormone; FT4: Free thyroxine.

**References**

1. Rizopoulos D. Joint models for longitudinal and time-to-event data: With applications in R: CRC press; 2012.

2. Fitzmaurice GM, Laird NM, Ware JH. Applied longitudinal analysis: John Wiley & Sons; 2012.

3. Lin H, Zelterman D. Modeling survival data: extending the Cox model. Taylor & Francis; 2002.

4. Derakhshan A, Sardarinia M, Khalili D, Momenan AA, Azizi F, Hadaegh F. Sex specific incidence rates of type 2 diabetes and its risk factors over 9 years of follow-up: Tehran Lipid and Glucose Study. PloS one. 2014;9(7):e102563.

5. Mchunu NN, Mwambi HG, Rizopoulos D, Reddy T, Yende-Zuma N. Using joint models to study the association between CD4 count and the risk of death in TB/HIV data. BMC Medical Research Methodology. 2022;22(1):1-9.

6. Illipse M, Czene K, Hall P, Humphreys K. Studying the association between longitudinal mammographic density measurements and breast cancer risk: a joint modelling approach. Breast Cancer Research. 2023;25(1):1-11.

7. Mauff K, Steyerberg EW, Nijpels G, van der Heijden AA, Rizopoulos D. Extension of the association structure in joint models to include weighted cumulative effects. Statistics in medicine. 2017;36(23):3746-59.

8. Hickey GL, Philipson P, Jorgensen A, Kolamunnage-Dona R. joineRML: a joint model and software package for time-to-event and multivariate longitudinal outcomes. BMC medical research methodology. 2018;18:1-14.

9. Kang K, Pan D, Song X. A joint model for multivariate longitudinal and survival data to discover the conversion to Alzheimer's disease. Statistics in Medicine. 2022;41(2):356-73.
